# Fatal disseminated pyogenic infection due to hypermucoviscous hypervirulent Klebsiella pneumoniae: A case report and literature review

**DOI:** 10.1002/ccr3.4754

**Published:** 2021-09-21

**Authors:** Emad M. Salawati

**Affiliations:** ^1^ Department of Family Medicine Faculty of Medicine King Abdulaziz University Jeddah Saudi Arabia

**Keywords:** hypermucoviscous, Klebsiella pneumonia, sepsis

## Abstract

Hypervirulent Klebsiella pneumonia is becoming recognized globally and has been associated with serious sequelae including death. However, ethnicity and metastatic infections are characteristics for hypermucoviscous hypervirulent Klebsiella pneumoniae (hvKp) and should be rolled in/out by PCR and/or string test.

## Graphical abstract

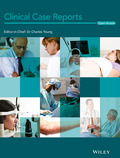

## 1 INTRODUCTION

Hypervirulent Klebsiella pneumoniae strains (hvKp) have been associated with liver abscesses and disseminated infections. Diabetes and being Asian are well‐known risk factors for hvKp. This is a case report of a 58‐year‐old diabetic male patient from the Philippines who presented with altered mental status and was admitted to the intensive care unit. Due to the patient's ethnicity and persistently positive blood culture, hvKp was suspected. A positive string test on a culture plate and CT scan results confirmed the diagnosis of hvKp in lung and liver abscesses. Incision and drainage of the liver abscess were planned but, unfortunately, the patient developed unexplained ventricular fibrillation and died.

Hypervirulent Klebsiella pneumoniae (hvKp) infection is becoming recognized globally as a possible cause of organ dysfunction or life‐threatening conditions and has emerged as a significant pathogen able to cause community‐acquired and, increasingly, hospital‐acquired infections in recent decades. Unlike the classic presentation, hvKp frequently causes infection in otherwise healthy people,^1^, ^2^, ^3^, ^4^ with prevalence ranging from 12% to 45% in endemic areas.^5^, ^6^, ^7^, ^8^, ^9^

In the late 1980s, cases reported in Taiwan outlined a distinct syndrome of community‐acquired Klebsiella pneumoniae infection. Pyogenic liver abscesses with metastasis were reported in healthy patients.^1^, ^10^, ^11^ Although these findings were first reported in the Asian Pacific Rim (e.g., Japan, Vietnam, Korea, and Taiwan), an increasing number of incidents have been reported in South and North America, the Caribbean, Europe, Australia, Africa and South Africa, and the Middle East.^12^, ^13^, ^14^, ^15^, ^16^, ^17^, ^18^, ^19^, ^20^, ^21^

Clinical and bacterial features distinguish the hvKp variant from classical strains. Essentially, the hvKp variant can cause serious infection in healthy individuals and unusual sites of infection, including endophthalmitis, meningitis, and necrotizing fasciitis. Furthermore, the variant has the ability to spread by metastasis. Finally, its appearance on agar plates is hypermucoviscous. This phenotype has been defined as a ‘string test’. The string test is positive when a bacteriology inoculation loop can generate a viscous string of more than 5 mm.^22^

An underlying immune‐suppressing medical condition can predispose a patient to the infection, including organ transplantation and diabetes mellitus.^17^ A global systematic survey reported that diabetes is the most prevalent predisposing medical condition of endogenous endophthalmitis. In addition, the most prevalent extraocular focus of infection is liver abscess.^18^

Even though most patients are young and healthy, the disease is still significantly associated with a high mortality rate, ranging from 3% to 42%. There have been remarkable mortality rates of 55% for community‐acquired pneumonia with bacteremia and 47% for necrotizing fasciitis. Furthermore, survivors with infection in critical sites frequently suffer disastrous morbidities such as blindness, neurologic sequelae, or loss of limbs.^22^

In this case report, a fatal case of hypermucoviscous hypervirulent Klebsiella pneumoniae (hvKp) in lung and liver abscesses was described. To the best of my knowledge, few studies in the Kingdom of Saudi Arabia or the Middle East have described the disease.

## 2 CASE REPORT

A 58‐year‐old diabetic male patient from the Philippines, not known to have any underlying medical conditions, was brought to the hospital due to altered mental status. On initial physical examination, his blood pressure was 80/50 mmHg despite fluid resuscitation, his heart rate was 120 beats per minute, his respiratory rate was 25/min, his temperature was 39°C, his oxygen saturation was 90%, and the Glasgow Coma Scale was 10. His respiratory examination revealed left lower lobe crackles, and the rest of the physical examination was unremarkable.

**FIGURE 1:**
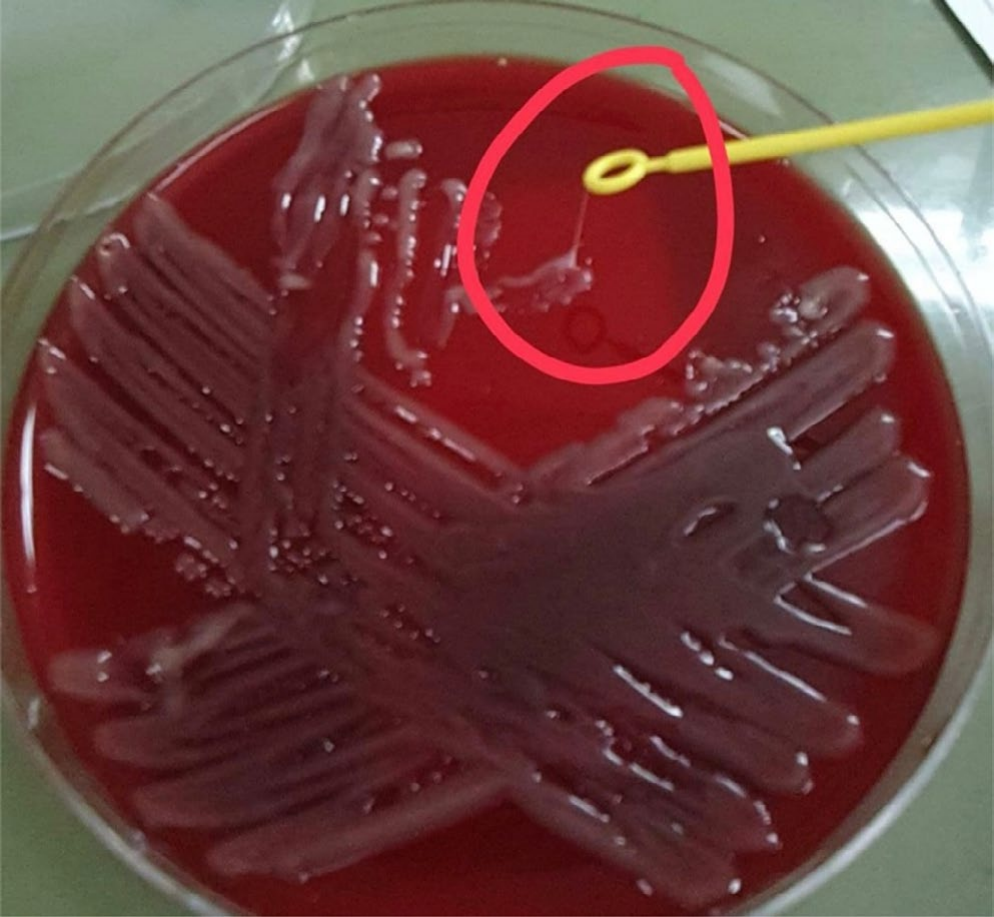
Positive string test on a Klebsiella pneumoniae hypervirulent strain from blood culture

**FIGURE 2:**
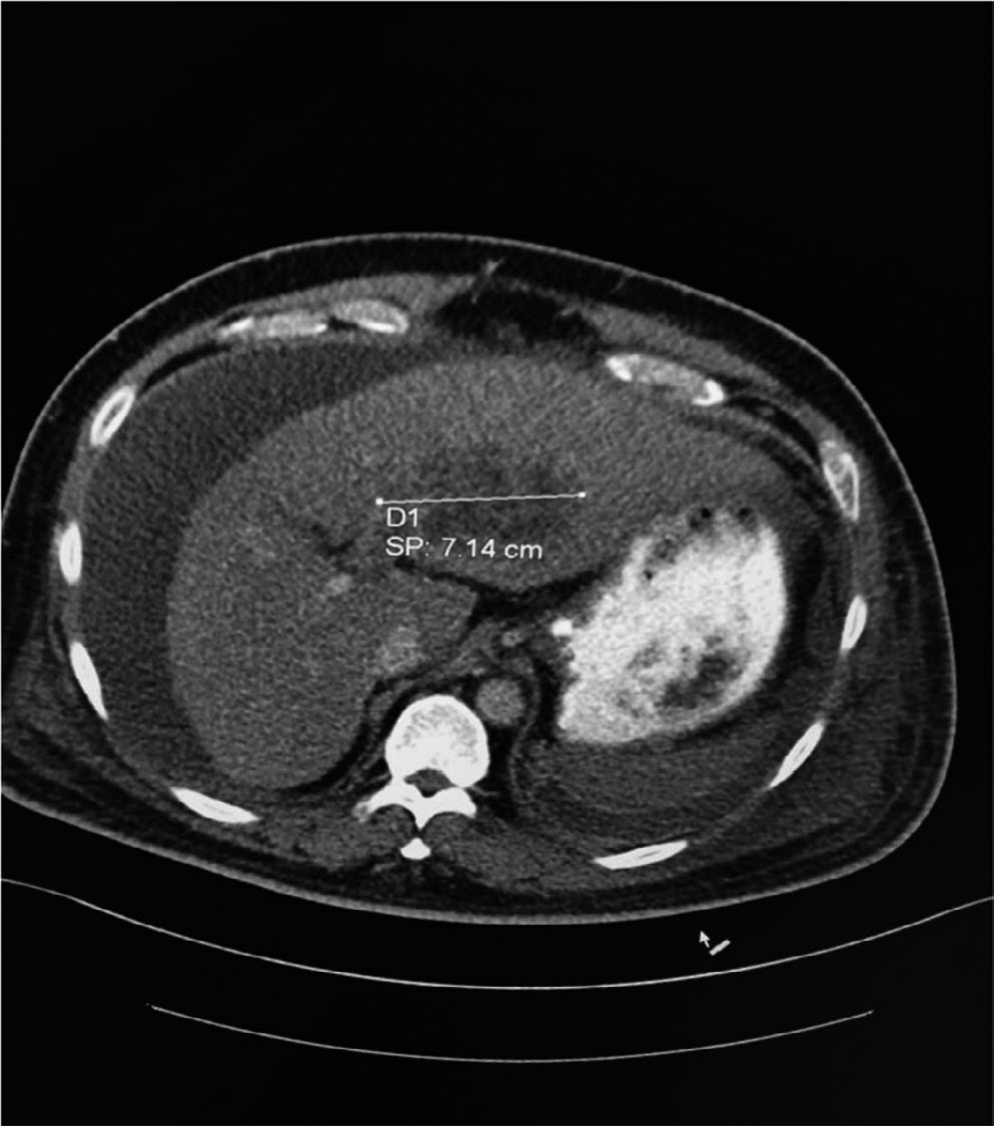
CT scan abdomen with contrast showed 7.14 cm multiloculated left liver lobe abscess

**FIGURE 3:**
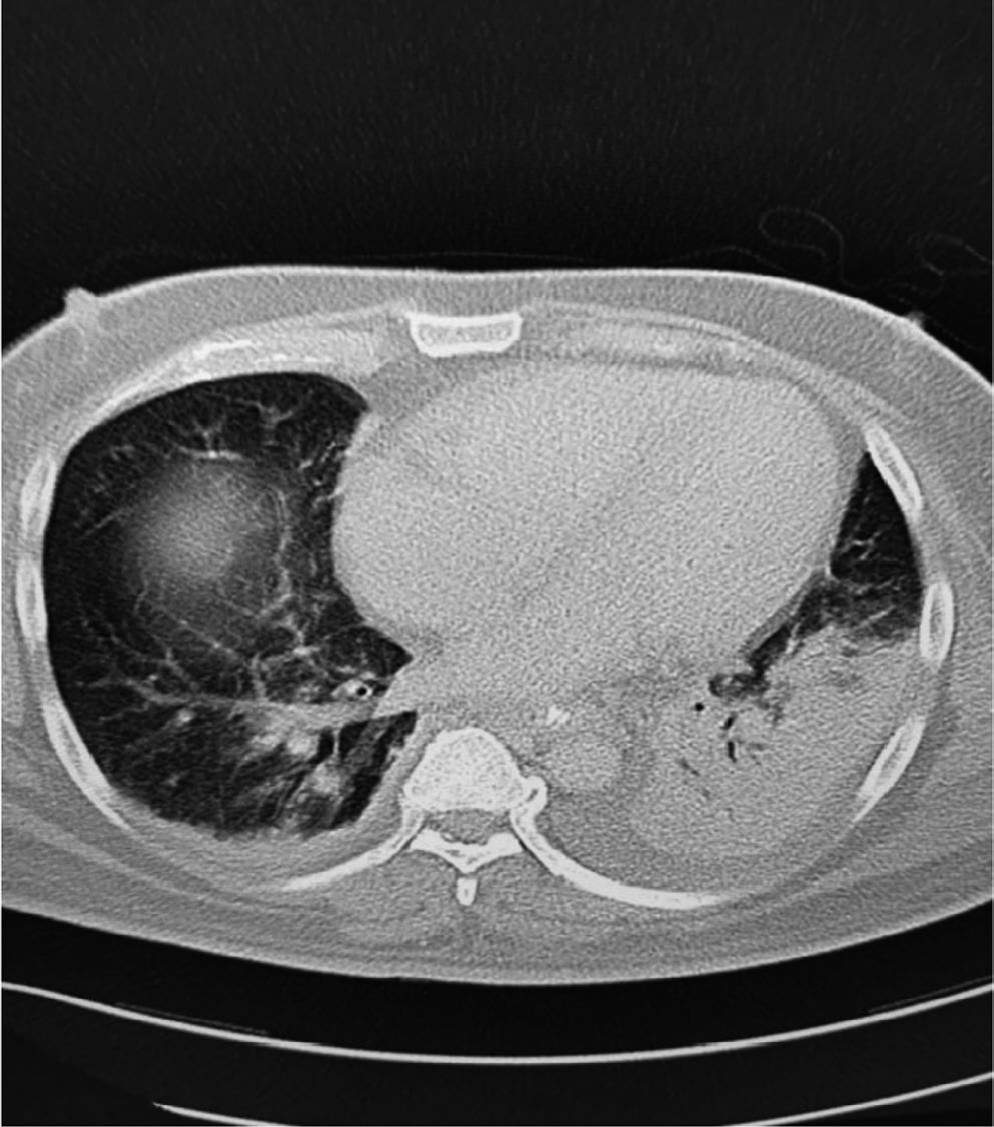
CT scan chest with contrast showed left lower lobe consolidation with air bronchogram

The basic laboratory investigation for a complete blood count (CBC) and baseline chemistry panel was positive for leukocytosis (WBCs was 20 × 10^9^/L) and acute kidney injury. The patient was admitted to the intensive care unit and started on intravenous vancomycin, meropenem, and norepinephrine. Later on, the blood culture was positive for pansensitive Klebsiella pneumoniae. Therefore, antibiotics were de‐escalated to IV ceftriaxone. The patient continued to be hypotensive, and repeated blood culture was persistently positive for pansensitive Klebsiella pneumoniae. Due to the patient's ethnicity and persistently positive blood culture, hypermucoviscous hypervirulent Klebsiella pneumoniae (hvKp) was suspected. The string test was positive on a culture plate (Figure 1). After stabilizing the patient, a pan CT scan was requested and showed evidence of disseminated pyogenic infection in the liver and left lung (Figures 2 and 3). Incision and drainage of the liver abscess were planned but, unfortunately, the patient developed unexplained ventricular fibrillation and died.

## 3 DISCUSSION

In this case report, we reported a fatal case of pulmonary and liver abscesses due to hvKp in a diabetic patient.

Recently, in Southeast Asia, a Klebsiella pneumoniae hypervirulent strain was recognized as an organism responsible for severe invasive infection and it exhibits hypermucoviscosity in string tests.^19^, ^20^, ^21^ A previous study in Taiwan reported an increased risk of complications of Klebsiella pneumoniae among patients with diabetes and liver abscess. These findings were also consistent with the findings reported in this case report.^11^ Moreover, a multiorgan involvement (brain, eye, and lung) case of embolic Klebsiella pneumoniae renal abscess was reported.^23^ The majority of Klebsiella pneumoniae infections result in pneumonia or urinary tract infections. However, over the last two decades, a unique, aggressive syndrome likely to cause liver abscesses has also been widely reported in Asia and it is emerging as a global disease, as demonstrated by the general awareness of the disease and the reported cases in the literature.^24^ Additionally, two cases of elderly diabetic patients with perinephric abscess causing Klebsiella pneumoniae endophthalmitis were described in Taiwan.^25^ Nevertheless, in Saudi Arabia, two cases of hepatic abscess causing Klebsiella pneumoniae endophthalmitis were reported.^26^ In contrast, in an animal model study, hvKp did not cause more severe infections than a nonhypermucoviscous strain.^27^ The use of carbapenem as the first‐line treatment of ESBL‐resistant hvKp endophthalmitis has a favorable outcome. Also, it has been hypothesized that hvKp obtains resistant and virulent plasmids via horizontal transmission, and that hvKp is mostly sensitive to most antimicrobial agents, but emerging resistance has been reported.^27^ Additionally, one report concluded that about 16% of extended‐spectrum beta‐lactamase (ESBL) produces hvKp.^28^

Based on the case's ethnicity and medical condition, hypermucoviscous hypervirulent Klebsiella pneumoniae was suspected and a CT scan was ordered. As previously mentioned, diabetic Asians are at a higher risk of contracting Klebsiella pneumoniae infection. Moreover, a case was reported of a diabetic patient from the Philippines presenting with three distant abscesses (liver and bilateral leg abscesses).^29^

When linked together, these case findings describe the disease. Early detection and treatment might result in a better outcome.

There is a gap in the knowledge and further research is required to address the worldwide increase in this infection, the impact of early diagnosis, and the best treatment modalities.

## 4 CONCLUSION

This case demonstrates the severity of hypermucoviscous hypervirulent Klebsiella pneumoniae, which can cause serious sequelae and mortality. Suspicion of hypermucoviscous hypervirulent K. pneumonia increased significantly due to the patient's ethnicity and diabetes. The case also emphasizes the significant contribution of diagnostic modalities, which enabled early detection using blood culture in conjunction with a string test and timely management. However, late presentation might end with unfortunate events.

## CONFLICT OF INTEREST

None declared.

## AUTHOR CONTRIBUTIONS

ES designed the study, manuscript writing, literature search, and manuscript revision.

## Data Availability

No further data are available.
